# Phase‐specific networks of job stress, anxiety and insomnia during acute crisis and chronic strain: A citywide study of public health nurses in Shanghai

**DOI:** 10.1002/gps3.70064

**Published:** 2026-09-29

**Authors:** Quyige Gao, Fan Hu, Ruijie Chang, Shangbin Liu, Chen Xu, Xin Ge, Birong Wu, Shunyu Tao, Yujie Liu, Rui Li, Lin Wang, Jun Ma, Yong Cai

**Affiliations:** ^1^ Institute of Clinical Research Tongren Hospital, Shanghai Jiao Tong University School of Medicine Shanghai China; ^2^ School of Public Health Shanghai Jiao Tong University School of Medicine Shanghai China; ^3^ Department of Nursing Shanghai Pulmonary Hospital Shanghai China; ^4^ Shanghai Jiao Tong University School of Nursing Shanghai China; ^5^ Center for Community Health Care Shanghai Jiao Tong University China Hospital Development Institute Shanghai China

**Keywords:** emergency preparedness, job stress, network analysis, public health nurses, workforce resilience

## Abstract

**Background:**

Psychological distress among public health nurses (PHNs) during major public health emergencies may vary across response phases, from acute crisis to prolonged chronic strain. Although the mental health consequences of sustained emergency response are well documented, evidence on how symptom connectivity patterns differ across acute and chronic phases remains limited.

**Aims:**

This study aims to compare symptom centrality and connectivity patterns of job stress, anxiety and insomnia among PHNs during acute crisis and chronic strain and to identify phase‐specific associations that may inform tailored intervention strategies.

**Methods:**

We conducted a city‐wide, two‐wave census survey of PHNs across all 249 community health centres in Shanghai, China. Data were collected during the acute crisis phase (October–November 2022; *n* = 1565) and the chronic strain phase (November 2023–January 2024; *n* = 1718), with response rates exceeding 96% at both waves. Validated instruments were used to assess job stress, anxiety and insomnia. Undirected symptom networks were estimated using graphical least absolute shrinkage and selection operator models to characterise symptom connectivity patterns, whereas Bayesian network modelling was used to explore conditional probabilistic dependencies among symptoms. Exploratory subgroup analyses examined network heterogeneity by age and working hours.

**Results:**

Overall psychological distress was lower during the chronic strain phase than during the acute crisis phase; however, symptom‐level connectivity patterns differed between the two phases, with notable differences in centrality. Conflicting responsibilities and limited opportunity to draw on prior experience and training showed stable metric‐specific centrality across both phases. During the acute phase, network centrality was anchored by operational instability—including unclear roles and high effort demands—alongside anxiety‐related hyperarousal. During chronic strain, the most central nodes were characterised by sustained workload burden and impaired recovery, such as difficulty relaxing. Bayesian models consistently positioned work‐related stressors at upstream positions within the distress architecture. Subgroup analyses revealed that symptoms were more tightly interconnected among younger PHNs (< 36 years) during acute crisis.

**Conclusions:**

The psychological architecture of workforce distress is phase‐specific; therefore, ‘one‐size‐fits‐all’ interventions are inadequate. Interventions should prioritise operational clarity during acute crises and workload management and recovery support during prolonged strain. Integrating these phase‐adapted strategies into public health systems may help sustain a resilient frontline workforce.

## Abstract (highlights)

**WHAT IS ALREADY KNOWN ON THIS TOPIC**

- Psychological distress is common among healthcare workers during major public health emergencies. Existing studies have predominantly focused on hospital‐based staff during acute outbreaks, and evidence regarding public health nurses (PHNs) working in community settings remains limited.

**WHAT THIS STUDY ADDS**

- This city‐wide repeated cross‐sectional study examines the phase‐specific differences in mental health symptom networks among PHNs during acute crisis and chronic strain. Network analyses revealed both stable and phase‐specific centrality patterns: conflicting responsibilities and limited opportunity to draw on prior experience and training showed stable metric‐specific centrality across both phases. Effort demands and anxiety‐related hyperarousal were more prominent in the acute crisis, whereas sustained workload burden and impaired recovery were more prominent in the chronic strain. In exploratory Bayesian network models, work‐related stressors frequently occupied upstream positions within the estimated conditional dependency structures. Furthermore, exploratory analyses revealed distinct age‐related network heterogeneity during the acute crisis phase, with younger PHNs (< 36 years) demonstrating more closely coupled symptom relations than their older peers.

**HOW THIS STUDY MIGHT AFFECT RESEARCH, PRACTICE OR POLICY**

- The findings suggest that symptom centrality patterns of psychological distress among PHNs are phase‐specific rather than uniform. Interventions during acute emergencies should prioritise role clarity and operational coordination, with additional targeted support for younger frontline personnel, who showed a more tightly coupled symptom network in exploratory subgroup analyses. During prolonged periods of strain, targeted workload management and recovery‐oriented strategies should be prioritised. Phase‐adapted approaches may improve workforce sustainability during future public health crises.

## INTRODUCTION

Recurrent large‐scale infectious disease outbreaks over the past 5 years have exposed persistent vulnerabilities in global public health infrastructure, placing unprecedented and sustained demands on community health services. This emergency context triggers a surge in service demand that disproportionately affects frontline professionals, particularly public health nurses (PHNs).^1^ As the backbone of the community response, PHNs have faced significant occupational stress, anxiety and sleep disturbances during pandemics such as Coronavirus Disease 2019 (COVID‐19).^2^, ^3^ Despite their indispensable role, PHN services remain chronically underfunded; the minimum standard ratio of 1 PHN to a population of 5000, as recommended by the U.S. Association of State and Territorial Directors of Nursing, remains unmet in many systems, resulting in persistently high workloads and cumulative psychological strain.^4^

Crucially, the psychological burden experienced by PHNs is not uniform across phases; it differs between an acute crisis phase and a subsequent period of chronic strain. The acute phase is characterised by operational urgency, with PHNs performing contact tracing and risk screening under conditions of high uncertainty and resource scarcity.^5^ These demands are often accompanied by intensified workloads and greater vulnerability to anxiety, insomnia and emotional exhaustion.^6^ During the chronic strain phase, operational chaos may subside, yet the cumulative effects of prolonged mobilisation often become more apparent.^7^ Although the prevalence of distress during these phases is documented, less is known about how symptoms are interconnected within each phase. Specifically, evidence on how symptom centrality and connectivity patterns differ across these distinct phases is scarce, limiting the development of phase‐sensitive support interventions.

Current literature suggests that such distress is driven by a syndemic interplay of job stress, anxiety and insomnia.^3^, ^6^ These factors exhibit complex, bidirectional relationships: job stress is a robust predictor of insomnia, while anxiety can trigger sleep disturbances that, in turn, amplify stress loops.^8^, ^9^ However, treating these relationships as uniform across contexts oversimplifies their complexity.^10^ Most existing studies rely on pairwise associations, failing to capture the overall structure of the symptom system.^11^ To address this gap, network analysis offers a methodological framework to conceptualise symptoms as interconnected components of a psychological system. This approach can identify central symptoms that sustain distress and bridge symptoms that facilitate contagion across domains.^12^, ^13^ Furthermore, Bayesian network models can describe conditional probabilistic dependencies among symptoms and provide an exploratory complement to undirected networks, although such models cannot establish causal ordering in cross‐sectional data.

Despite the high prevalence of distress among PHNs,^14^ the phase‐specific organisation of job stress, anxiety and insomnia symptom networks remains unmapped.^15^ This study aims to address this gap by (1) comparing the network characteristics of these symptoms among PHNs during the acute crisis and chronic strain phases; (2) identifying phase‐specific central and bridge symptoms and (3) exploring conditional dependency patterns among symptoms using Bayesian network modelling. By characterising these phase‐specific connectivity patterns, we seek to inform the design of targeted phase‐sensitive strategies to support the mental health and functional capacity of this vital workforce during prolonged public health emergencies.

## METHODS

### Study design and setting

We conducted a census‐based, two‐wave cross‐sectional study of the entire PHN workforce across all 249 community health centres in Shanghai. This design allowed for a comprehensive assessment of the workforce during two distinct epidemiological phases.

Wave 1 (acute crisis phase): Data were collected from October to November 2022. This period coincided with a critical juncture of rapid viral transmission and the enforcement of stringent, citywide containment measures, representing a peak in operational urgency and uncertainty.

Wave 2 (chronic strain phase): The second wave was conducted approximately one year later, from November 2023 to January 2024. By this stage, although emergency operations had de‐escalated, the workforce faced the cumulative sequelae of prolonged mobilisation and the resumption of routine public health duties.

Data were collected through the secure online platform SurveyStar (Changsha Ranxing Science and Technology, Shanghai, China), with support from municipal health authorities to facilitate access.

### Participants and procedures

The target population comprised all registered PHNs primarily engaged in preventive healthcare within the community health system. Recruitment was coordinated centrally by the Shanghai Health Promotion Centre, utilising a cascade strategy in which centre leaders distributed digital access codes to staff. To ensure data quality, the survey platform incorporated automated logic checks to prevent invalid or incomplete submissions. Strict anonymity was maintained; no personally identifiable information was recorded. Response rates were high in both waves: 96.7% (1565/1618) in wave 1 and 96.2% (1718/1785) in wave 2, reducing the risk of selection bias (figure 1).

**FIGURE 1:**
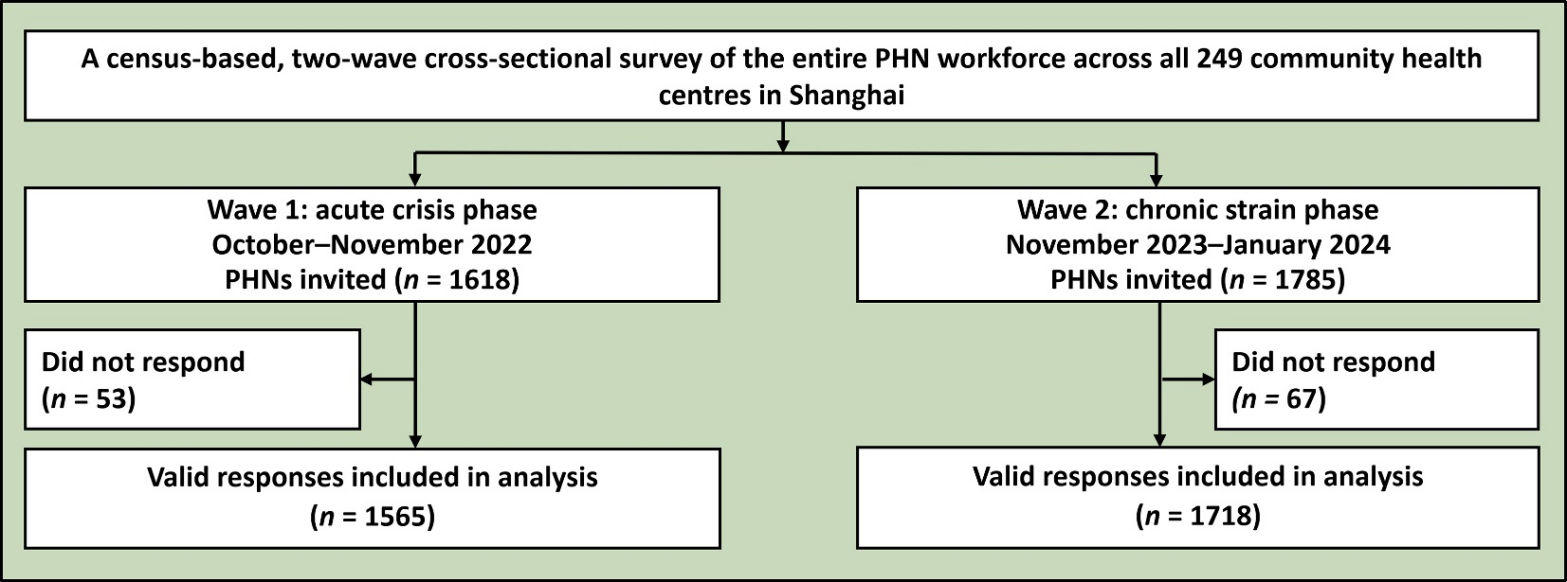
The flowchart of the participant enrolment procedure. PHN, public health nurse.

### Measures

#### Sociodemographic and occupational profile

We collected data on key demographic variables (sex, age, marital status and educational level) and occupational characteristics (professional title grade, institutional tenure and daily working hours) to characterise the workforce.

#### Job stress

Job stress was assessed using the 13‐item Job Stress Questionnaire (JSQ‐13), which is based on Caplan's person–environment fit theory.^16^ The instrument captures four dimensions of occupational strain: workload (5 items), role conflict (2 items), role ambiguity (3 items) and skill utilisation (3 items), as detailed in Supporting Information S1: table S1. Items are rated on a 7‐point Likert scale. The scale demonstrated excellent internal consistency in the present study (Cronbach's *α*: Wave 1 = 0.911, Wave 2 = 0.917).

#### Anxiety symptoms

Anxiety severity was measured using the Generalised Anxiety Disorder‐7 (GAD‐7) scale.^17^ This widely validated tool assesses the frequency of anxiety symptoms over the preceding 2 weeks on a 4‐point scale. Total scores range from 0 to 21, with higher scores indicating greater symptom severity. The scale showed high internal consistency in our sample (Cronbach's *α*: Wave 1 = 0.971, Wave 2 = 0.969).

#### Insomnia

Insomnia symptoms were assessed using three items capturing difficulty initiating sleep, difficulty maintaining sleep and early‐morning awakening (Supporting Information S1: table S1).^18^ Participants rated each item on a 3‐point scale (1 = rarely or never, 2 = sometimes, 3 = nearly every night for more than 2 weeks), with higher total scores indicating greater symptom severity. The scale demonstrated good internal consistency in both waves (Cronbach's *α* = 0.872 and 0.857, respectively).

### Statistical analysis

#### Descriptive statistics

Descriptive analyses were performed using frequencies and percentages for categorical variables, and means with standard deviations (SD) for continuous variables. Independent samples *t*‐tests were used to compare job stress, anxiety and insomnia scores between the two waves (Supporting Information S1: table S2).

#### Network estimation

Psychometric networks were estimated separately for each wave using the ‘qgraph’ and ‘bootnet’ packages in R.^19^ Each node represented a single questionnaire item; the full item wording is provided in Supporting Information S1: table S1. Network estimation used the extended Bayesian information criterion graphical least absolute shrinkage and selection operator (EBICglasso), which regularises the inverse covariance matrix and shrinks negligible partial correlations to zero, thereby reducing spurious associations and improving interpretability. The optimal tuning parameter (*λ*) was selected via EBIC model comparison. Edges represent regularised partial correlations between node pairs, controlling for all remaining nodes. Edge thickness and colour indicate association strength and direction; network figures were produced using the ‘ggplot2’ package. To characterise topological properties, three global network metrics were computed for each wave: network density (the proportion of present edges among all possible edges), mean local clustering coefficient (the average tendency of a node's neighbours to be interconnected) and average path length (the mean shortest distance between node pairs, weighted by inverse edge strength). Given the 23‐node network, the sample size in each wave substantially exceeded the commonly recommended minimum of five to ten observations per estimated node for exploratory network estimation.^19^

#### Centrality and bridge analysis

To evaluate node importance, strength, betweenness and closeness centrality indices were computed for all nodes using the ‘qgraph’ package.^19^ Expected influence (EI) was additionally calculated to capture the signed sum of edge weights for each node, reflecting its overall signed connectivity within the network. Bridge EI (BEI) was computed using the ‘networktools’ package to identify nodes that most strongly link distinct symptom clusters, thereby quantifying cross‐domain connectivity.

#### Network stability

Network accuracy and stability were evaluated using the ‘bootnet’ package in R. Edge weight precision was assessed via nonparametric bootstrapping (1000 resamples), generating 95% confidence intervals. Centrality stability was examined through case‐dropping subset bootstrapping (1000 iterations), and the correlation stability (CS) coefficient was computed; according to the framework reported by Epskamp et al.,^19^ CS values above 0.25 indicate acceptable stability, whereas values above 0.50 indicate strong robustness. Bootstrap difference tests (*α* = 0.05) were additionally conducted to assess whether observed differences in edge weights and node strength were statistically distinguishable.^19^ Network comparison tests (NCTs; 1000 permutations) were performed to compare global network structure and strength between phases.

#### Bayesian network

Bayesian network models were constructed to explore conditional dependency structures among job stress, anxiety and insomnia items. Bayesian networks represent conditional dependencies between variables as directed acyclic graphs (DAGs), providing a structured framework for examining plausible hierarchical patterns of association.^20^ Given the repeated cross‐sectional design of the present study, these directed edges are considered strictly exploratory and do not support causal inference; they should be interpreted as conditional probabilistic associations rather than causal relationships. Models were estimated using the ‘bnlearn’ package with the Tabu search algorithm, selected for its robustness in high‐dimensional structure learning. All Bayesian network analyses were conducted using R version 4.3.3.

#### Exploratory subgroup analyses

Exploratory subgroup analyses were performed using NCTs to examine potential heterogeneity in network structure by age (median split) during both phases and by daily working hours (≤ 8 vs. > 8 hours) during the acute crisis phase only, owing to insufficient sample size in the > 8 hours group during the chronic strain phase (*n* = 106).

## RESULTS

### Participant characteristics and psychological outcomes

The study included 1565 PHNs during the acute crisis phase and 1718 during the chronic strain phase. Demographic characteristics were broadly comparable across waves, with no significant differences in sex distribution, marital status or place of origin (table 1). Mean age was similar (37.39 vs. 37.74 years; *t* = −1.34, df = 3222.50, *p* = 0.179). Reflecting the operational context of each phase, the distribution of professional titles differed between waves (*χ* ^2^ = 8.60, df = 3, *p* = 0.035), and wave 2 included a substantially larger proportion of nurses working standard hours (≤ 8 hours/day: 93.83% vs. 70.22%; *χ* ^2^ = 318.88, df = 2, *p* < 0.001).

**TABLE 1:** Sociodemographic and occupational characteristics of the study population by phase

	Acute crisis phase (*n* = 1565)	Chronic strain phase (*n* = 1718)	Statistic	df	*p* value
Sociodemographic characteristics					
Sex, *n* (%)					
Men	23 (1.47)	29 (1.69)	*χ* ^2^ = 0.13	1	0.718
Women	1542 (98.53)	1689 (98.31)			
Age (years), mean (SD)	37.39 (7.76)	37.74 (7.45)	*t* = −1.34	3222.50	0.179
Educational level, *n* (%)					
Below bachelor's degree	528 (33.74)	516 (30.03)	*χ* ^2^ = 5.01	1	0.025
Bachelor's degree or higher	1037 (66.26)	1202 (69.97)			
Marital status, *n* (%)					
Single	156 (9.97)	166 (9.66)	*χ* ^2^ = 0.09	2	0.956
Divorced or widowed	67 (4.28)	73 (4.25)			
Married or in a relationship	1342 (85.75)	1479 (86.09)			
Place of origin, *n* (%)					
Large city	1010 (64.54)	1118 (65.08)	*χ* ^2^ = 0.61	2	0.738
Small or medium‐sized city	225 (14.38)	231 (13.45)			
Rural	330 (21.09)	369 (21.48)			
Work‐related characteristics					
Professional rank, *n* (%)					
None	89 (5.69)	97 (5.65)	*χ* ^2^ = 8.60	3	0.035
Junior	681 (43.51)	664 (38.65)			
Middle	785 (50.16)	947 (55.12)			
Vice‐senior or above	10 (0.64)	10 (0.58)			
Daily working hours, *n* (%)					
≤ 8	1099 (70.22)	1612 (93.83)	*χ* ^2^ = 318.88	2	< 0.001
9–10	353 (22.56)	72 (4.19)			
> 10	113 (7.22)	34 (1.98)			
Institutional tenure (years), *n* (%)					
≤ 5	467 (29.84)	468 (27.24)	*χ* ^2^ = 2.81	2	0.246
6–15	676 (43.19)	762 (44.35)			
> 15	422 (26.96)	488 (28.41)			
Years in public health service, *n* (%)					
≤ 5	674 (43.07)	674 (39.23)	*χ* ^2^ = 5.46	2	0.065
6–15	757 (48.37)	875 (50.93)			
> 15	134 (8.56)	169 (9.84)			

Abbreviation: SD, standard deviation.

Mean job stress scores were higher in the acute crisis phase than in the chronic strain phase (45.13 [13.34] vs. 41.84 [13.12]), as were mean GAD‐7 scores (5.87 [5.15] vs. 4.47 [4.61]) (both *p* < 0.001). Mean scores for all three insomnia items were also lower in the chronic strain phase (Supporting Information S1: table S2). Nonetheless, symptoms remained present across both waves, indicating that distress persisted at a lower level rather than resolving entirely.

### Topological architecture of distress networks

The distress networks exhibited a moderately dense global structure across both phases (figure 2), with comparable density (0.459 vs. 0.463), a higher clustering coefficient (0.601 vs. 0.637) and a longer average path length (22.530 vs. 27.042), suggesting greater within‐domain consolidation and less direct cross‐domain connectivity in the chronic strain phase. Global network structure did not differ significantly between phases (NCT maximum edge‐weight difference statistic, *M* = 0.150, *p* = 0.146).

**FIGURE 2:**
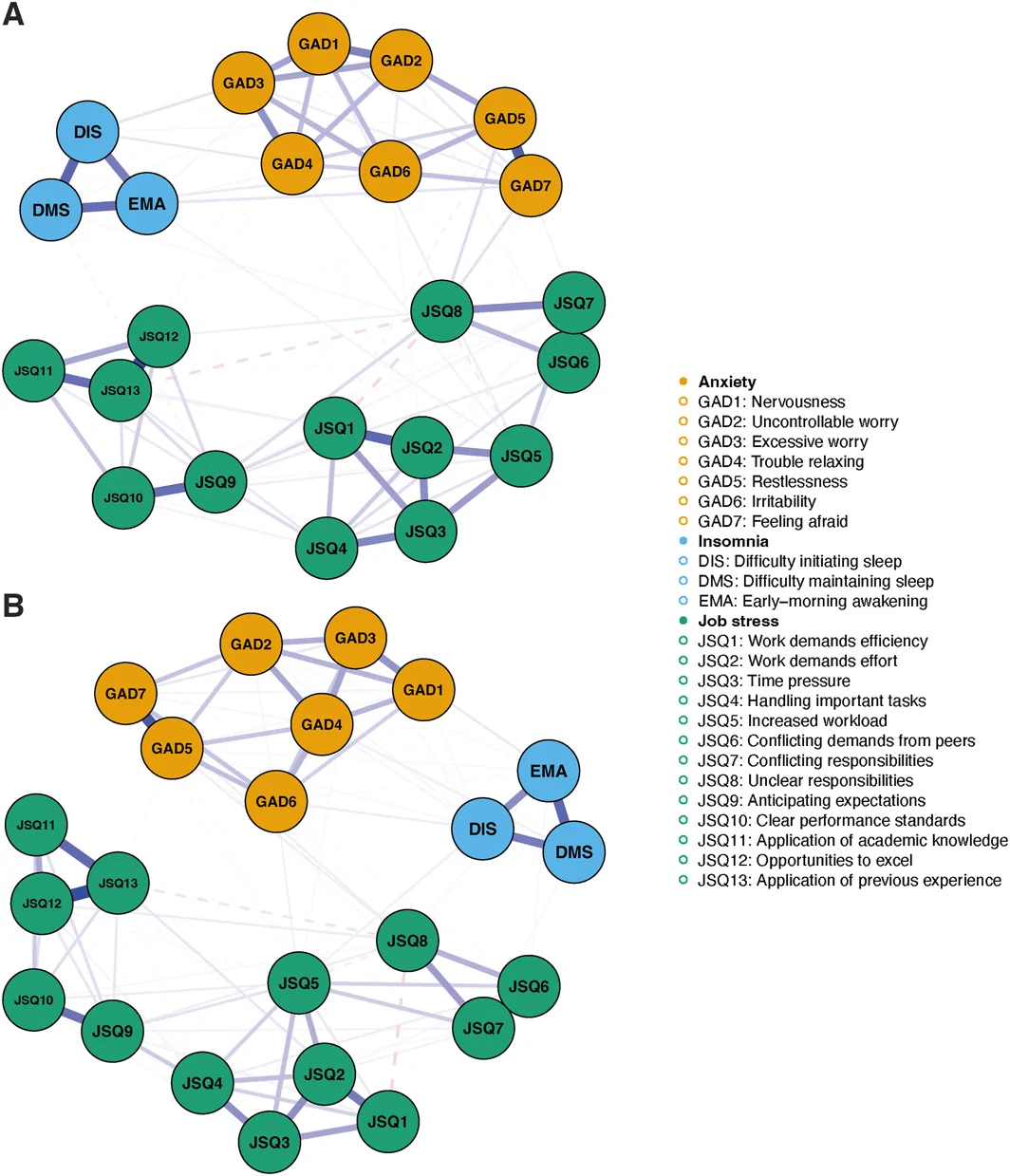
Undirected networks of job stress, anxiety and insomnia during the (A) acute crisis and (B) chronic strain phases. Each edge represents a partial correlation between two items, with positive correlations indicated in blue and negative correlations in red. The maximum correlation magnitudes are 0.642 (A) and 0.680 (B). The thickness of the edges reflects the absolute value of the correlation. Node colours represent different communities identified within the network: job stress (green), anxiety (orange) and insomnia (blue). Abbreviations for the item labels are provided on the right side of the figure. DIS, difficulty initiating sleep; DMS, difficulty maintaining sleep; EMA, early‐morning awakening; GAD, generalised anxiety disorder; JSQ, job stress questionnaire.

Both networks exhibited strong within‐domain cohesion, with the strongest edges consistently observed within rather than between symptom clusters. The edge between ‘Conflicting demands from peers’ (JSQ6) and ‘Conflicting responsibilities’ (JSQ7) was the strongest connection in both waves (weights: 0.642 and 0.680, respectively).

Subtle differences emerged in secondary connections. During the acute crisis phase, relatively strong associations were observed between anxiety‐related items (e.g., ‘Restlessness’–‘Feeling afraid’, weight = 0.413) and between sleep onset items. During the chronic strain phase, stronger associations emerged within the professional competency domain (e.g., ‘Opportunities to excel’–‘Application of previous experience’, weight = 0.527).

### Phase‐specific centrality patterns

Centrality analysis identified both stable and phase‐specific patterns of node importance (figure 3). Two job‐stress items showed stable but metric‐specific centrality across both waves: ‘application of previous experience’ (JSQ13) consistently showed the highest strength centrality (figure 3, Supporting Information S1: figure S1), whereas ‘conflicting responsibilities’ (JSQ7) consistently showed the highest EI (Supporting Information S1: table S4). Complete 23 × 23 edge weight matrices are reported in Supporting Information S1: table S3, with corresponding heatmap visualisations provided in Supporting Information S1: figure S2.

**FIGURE 3:**
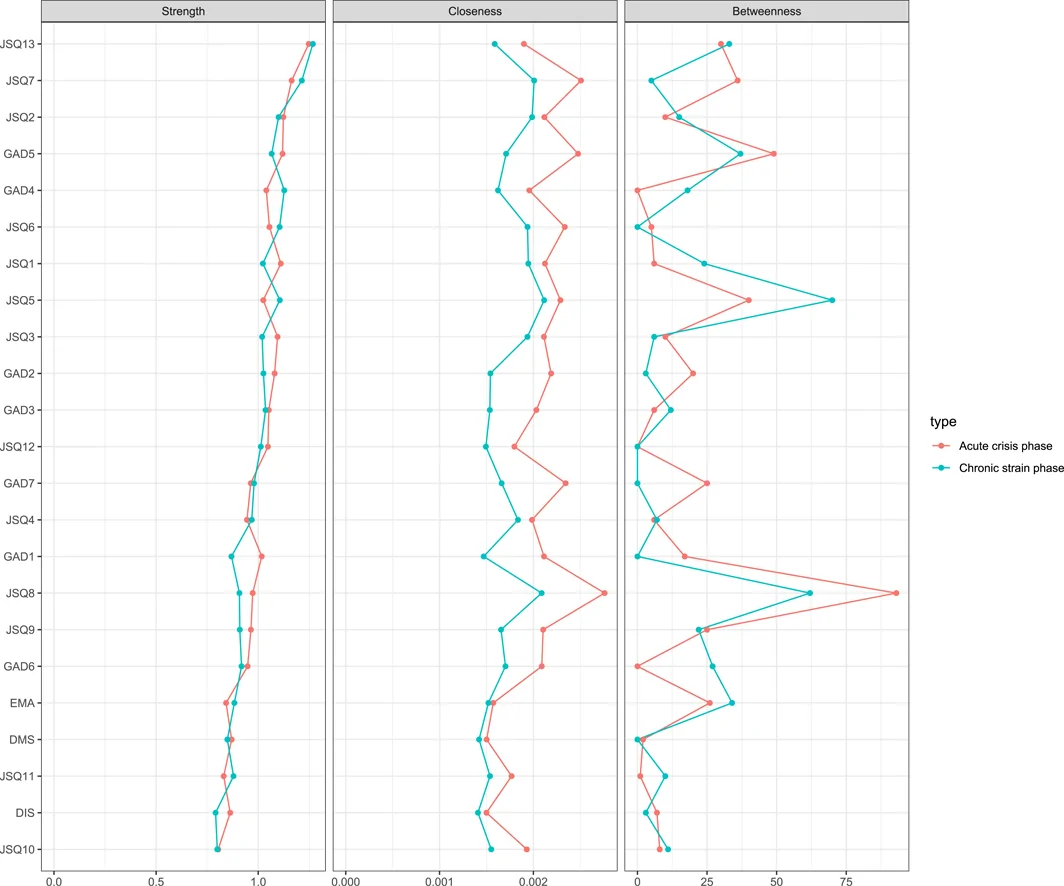
Node centrality metrics of the network during the acute crisis and chronic strain phases. The left, centre and right panels display the strength, closeness and betweenness values for each node, respectively. DIS, difficulty initiating sleep; DMS, difficulty maintaining sleep; EMA, early‐morning awakening; GAD, generalised anxiety disorder; JSQ, job stress questionnaire.

Beyond this stable core, the relative centrality of other nodes differed across phases. During the acute crisis phase, ‘Work demands effort’ (JSQ2) and ‘Restlessness’ (GAD5) showed elevated centrality (figure 3, Supporting Information S1: figure S1). During the chronic strain phase, ‘Increased workload’ (JSQ5) and ‘Trouble relaxing’ (GAD4) showed comparatively higher centrality. These differences in centrality profiles suggest that the nodes most strongly embedded in the symptom network differed between the acute and chronic phases.

### Bridge symptoms across phases

BEI analysis identified nodes linking the three symptom domains (figure 4; Supporting Information S1: table S4). Within the insomnia domain, the node with the highest bridge centrality differed by phase. During the acute crisis, ‘difficulty initiating sleep’ had the highest BEI (0.157); during chronic strain, ‘early‐morning awakening’ had the highest BEI (0.153), indicating a phase‐specific shift in the insomnia symptom with the strongest cross‐domain connectivity.

**FIGURE 4:**
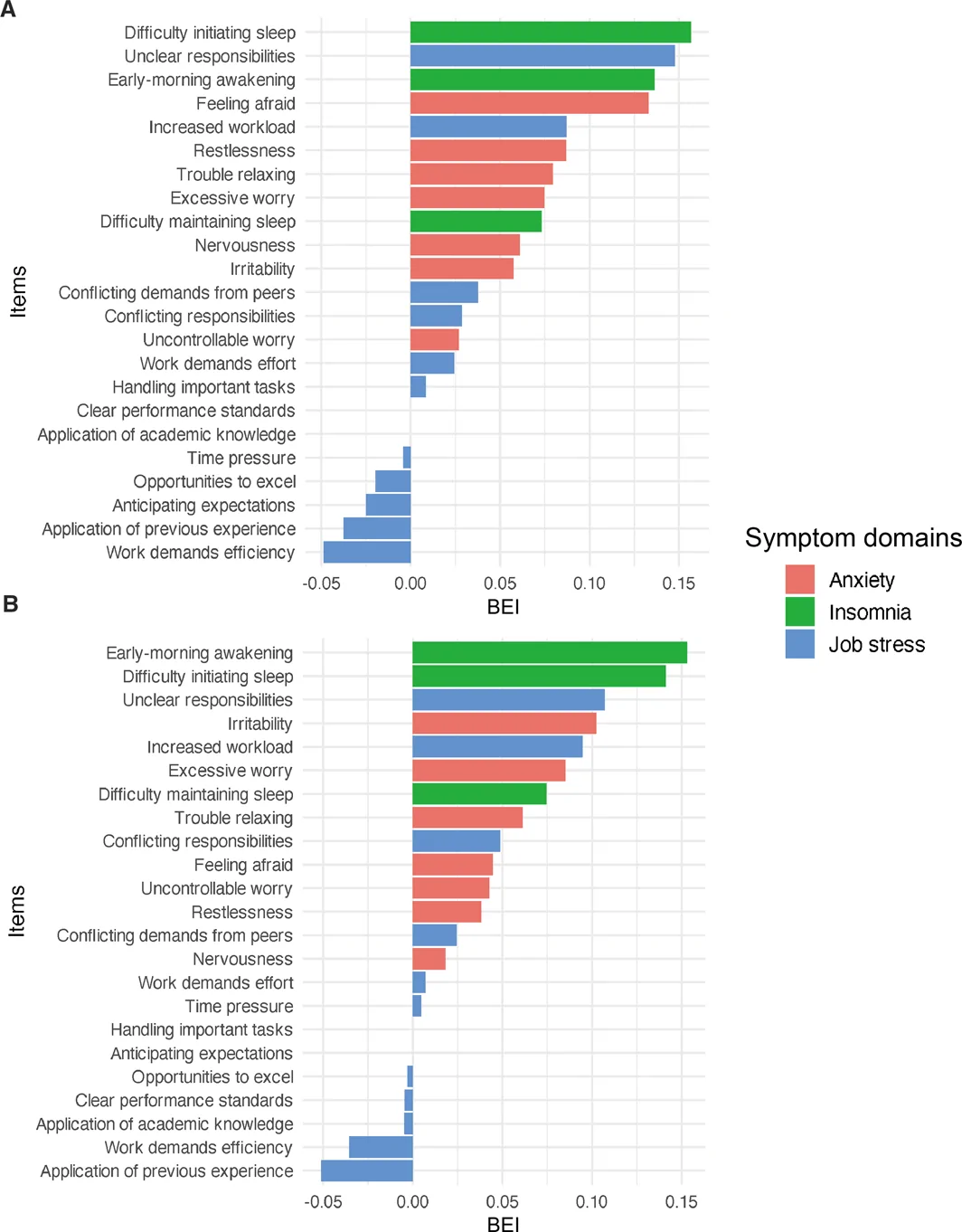
BEI values for job stress, anxiety and insomnia symptoms during the (A) acute crisis and (B) chronic strain phases. BEI, Bridge expected influence.

### Network stability and robustness

CS coefficients for node strength were 0.75 in both waves, exceeding the recommended threshold of 0.50 and indicating robust centrality stability (figure 5). Bootstrap confidence intervals indicated that most non‐zero edges were estimated with reasonable precision, and bootstrap‐based difference tests identified statistically distinguishable differences in selected edge weights across phases (Supporting Information S1: figures S1–S5).

**FIGURE 5:**
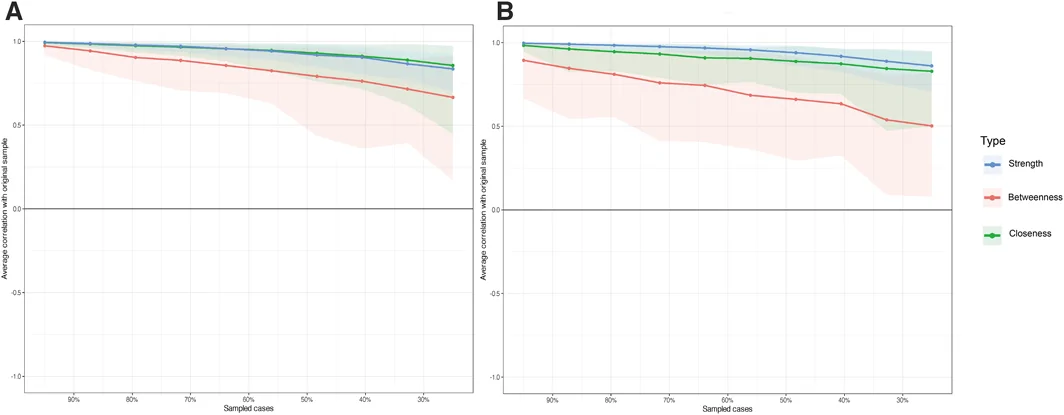
Stability of centrality metrics during the (A) acute crisis and (B) chronic strain phases. Point estimates and corresponding 95% confidence intervals of centrality metrics were assessed using a case‐drop bootstrap procedure. Stability was evaluated based on the average correlations between centrality indices derived from subsamples with randomly excluded nurses and those from the full dataset. Solid lines represent mean estimates, and shaded areas denote the 2.5th–97.5th percentile range.

### Exploratory directional associations: Bayesian network analysis

Bayesian network analysis was conducted as an exploratory complement to the undirected network models, characterising conditional dependency structures among symptom domains (Supporting Information S1: figure S6). In the estimated DAGs for both phases, job stress items frequently occupied upstream positions. This pattern indicates a stable conditional dependency structure involving occupational stress, anxiety and insomnia symptoms, but should not be interpreted as evidence of temporal or causal priority.

### Exploratory subgroup analyses

To examine potential heterogeneity by key occupational characteristics, exploratory subgroup analyses were performed using NCTs (Supporting Information S1: table S5; Supporting Information S1: figures S7 and S8). During the acute crisis, younger PHNs (< 36 years, *n* = 733) exhibited a significantly stronger global network than their older peers (≥ 36 years, *n* = 832; global strength difference statistic, *S* = 0.692, *p* = 0.021), although the overall network structure did not differ significantly (*M* = 0.160, *p* = 0.502).

Younger PHNs showed a higher network density (0.443 for < 36 years vs. 0.407 for ≥ 36 years) and a shorter average path length (20.790 for < 36 years vs. 24.960 for ≥ 36 years), suggesting more closely coupled symptom relations. Conversely, older PHNs displayed a higher clustering coefficient (0.585 for ≥ 36 years vs. 0.556 for < 36 years), indicating more localised symptom clustering. No other significant differences in network strength or structure were observed by working hours during acute crisis or by age during chronic strain (all *p* > 0.05; Supporting Information S1: table S5). Due to the small sample size (*n* = 106), the working‐hours subgroup analysis was restricted to the acute phase.

## DISCUSSION

### Main findings

To our knowledge, this is the first census‐based, two‐wave cross‐sectional study to examine phase‐specific differences in the centrality and connectivity of job stress, anxiety and insomnia symptoms among PHNs during acute crisis and chronic strain. By integrating undirected network analysis with Bayesian modelling, we identified a consistent pattern: although overall distress was lower during the chronic strain phase and global network structure did not differ significantly between phases, symptom centrality showed distinct phase‐specific differences. Specifically, a stable core of job‐related stressors persisted across phases, while node centrality differed between phases: acute operational demands and anxiety‐related hyperarousal were more central during acute crisis, and sustained workload burden and impaired recovery were more central during chronic strain. These findings challenge static models of occupational distress and underscore the need for adaptive, phase‐sensitive support strategies.

Across both epidemiological phases, conflicting responsibilities and limited opportunity to draw on prior experience and training emerged as stable job‐stress anchors within the distress network. This pattern suggests that some structural stressors in PHN work may remain salient across emergency contexts. Drawing on Role Theory, this persistence likely reflects the inherent tension in the PHN role: the dual demand to execute routine preventive care while simultaneously managing emergency response creates a state of chronic role conflict.^21^ Furthermore, the consistent centrality of ‘application of previous experience’ may reflect a mismatch between nurses' existing skills and the demands of novel emergency tasks. This mismatch may be associated with reduced professional self‐efficacy and greater anxiety‐related distress across phases.^22^ These findings suggest that role clarification and competency training may be important across phases, rather than being confined to the acute response.

Our analysis showed phase‐specific differences in centrality patterns. During acute crisis, the network was dominated by ‘work demands effort’ and ‘restlessness’, reflecting a pattern consistent with heightened cognitive and physiological activation during urgent response work. This aligns with cognitive load theory,^23^ which suggests that the volume of rapid decision‐making and information processing during acute crisis may exceed cognitive capacity and manifest as hyperarousal.^24^, ^25^ During chronic strain, ‘increased workload’ and ‘trouble relaxing’ were prominent nodes. This contrast is consistent with theoretical distinctions between an acute ‘fight‐or‐flight’ response and allostatic load, the cumulative physiological burden associated with chronic overactivation.^26^ The prominence of ‘trouble relaxing’ during chronic strain suggests that the primary issue is no longer just the stressor itself, but rather difficulties in post‐work recovery and deactivation.^27^ This distinction is clinically vital: acute phase interventions may focus on ‘reducing the load’ (operational streamlining), whereas chronic phase interventions may focus on ‘facilitating recovery’ (mandatory rest, detachment strategies).^28^

A potentially informative finding was the phase‐specific difference in bridge symptoms connecting anxiety to insomnia. During acute crisis, ‘difficulty initiating sleep’ was the primary bridge, a symptom often associated with anxiety‐related hyperarousal.^29^ This may reflect work‐related rumination at bedtime, which can interfere with sleep onset.^30^ During chronic strain, however, the primary bridge was early‐morning awakening. Previous studies have associated early‐morning awakening with depressive symptoms and exhaustion‐related states.^31^, ^32^ This difference may indicate distinct sleep‐related correlates of distress across phases, although the present design cannot identify the underlying physiological mechanisms.

Bayesian network analysis provided an exploratory conditional‐dependency perspective, with job stress items consistently occupying upstream positions in the estimated DAGs. This pattern indicates stronger probabilistic dependencies between occupational stress and anxiety/insomnia symptoms than in the reverse direction, although these directed edges represent statistical dependencies derived from cross‐sectional data and should not be interpreted as causal.^33^ This exploratory finding aligns with the job demands‐resources model's proposition that excessive demands are associated with impaired health.^34^ The consistency of this hierarchy across both waves suggests that occupational stress may warrant priority in intervention design, although symptom‐focused interventions targeting anxiety or sleep alone may be insufficient if occupational stressors remain unaddressed.

Meanwhile, the exploratory finding that younger PHNs had a denser and more strongly interconnected distress network during acute crisis suggests that early‐career PHNs may be more vulnerable to coordinated symptom activation. This aligns with the stress‐vulnerability hypothesis, whereby limited coping resources and professional experience may be associated with a more tightly interconnected symptom network.^35^, ^36^ Katsuta et al. reported that younger and newer healthcare workers in Japan had the highest rates of depression during the COVID‐19 pandemic, irrespective of occupation,^35^ whereas Lorente et al. found that nurses with insufficient preparation and limited coping resources were more likely to experience psychological distress when exposed to acute occupational stressors.^36^ These findings imply that phase‐sensitive interventions may also need to account for age and professional experience, with additional proactive support for younger nurses during acute emergencies.

### Limitations

Our findings should be interpreted in light of several limitations. First, because of substantial workforce turnover during the interval between the crisis and post‐emergency phases, a strictly paired longitudinal design was not feasible, and a repeated cross‐sectional approach was adopted. Although census‐based sampling in both waves enhances population representativeness at each time point, the possibility of a healthy worker effect cannot be excluded, whereby individuals with more severe psychological distress may have exited the workforce prior to wave 2. Second, although Bayesian network analysis provides probabilistic insights into symptom interrelationships, the cross‐sectional nature of the data precludes definitive causal inference or temporal ordering. Accordingly, the directed pathways identified should be interpreted as exploratory rather than causal. Third, reliance on self‐reported measures may introduce recall bias and common method variance, and residual confounding by unmeasured factors (e.g., organisational or personal resources) remains possible. Finally, as this study was conducted in a highly resourced urban setting, the generalisability of the findings to other healthcare contexts may be limited.

### Implications

The phase‐specific nature of these findings highlights the need for phase‐sensitive interventions at both organisational and individual levels. During acute crises, health systems should establish standardised emergency protocols and deploy digital triage to reduce role ambiguity, whereas peer coaching and psychological first aid may help address acute anxiety at the individual level.^37^ During prolonged periods of strain, flexible scheduling, mandatory rotation of high‐intensity duties and mental health training for managers may help limit fatigue accumulation and enable the early recognition of exhaustion; structured rest periods and sleep hygiene programmes may also help address cumulative recovery deficits. Incorporating these strategies into national preparedness plans—akin to Singapore's ‘Psychological Preparedness Toolkit’^38^—may be crucial for workforce resilience.

In conclusion, symptom centrality patterns among PHNs differed across phases, with anxiety‐related hyperarousal more prominent during acute crisis and recovery‐related symptoms more prominent during chronic strain. Recognising this phase‐specific contrast is key to protecting the workforce through targeted, phase‐specific interventions.

## AUTHOR CONTRIBUTIONS

Quyige Gao and Fan Hu were responsible for data curation, formal analysis, visualisation and drafting the original manuscript. Rui Li, Lin Wang, Jun Ma and Yong Cai were responsible for funding acquisition, resource coordination, resource provision, supervision and critical manuscript review and editing. Ruijie Chang, Shangbin Liu, Chen Xu and Xin Ge were responsible for project administration, software development and validation. Birong Wu, Shunyu Tao and Yujie Liu contributed to conceptualisation, investigation and methodology development. All authors had full access to all the data in the study, verified the accuracy and integrity of the data and approved the final version of the manuscript for submission.

## FUNDING

This study was supported by the Key Discipline Projects of the Shanghai Three‐Year Action Plan for Public Health (GWVI‐11.1‐29), the National Natural Science Foundation of China (Grant No. 72674180), the Public Health Research Project from the Shanghai Disease Control and Prevention Administration (2024GKM30) and the Key Project of Medical‐Engineering Interdisciplinary Programme, Shanghai Jiao Tong University (AF7320007, YG2025ZD29).

## CONFLICT OF INTEREST STATEMENT

The authors declare no conflicts of interest.

## ETHICS STATEMENT

The study protocol was approved by the Ethics Review Committee of the School of Public Health, Shanghai Jiao Tong University School of Medicine (Approval No.: SJUPN‐202108). The authors assert that all procedures contributing to this work comply with the ethical standards of the relevant national and institutional committees on human experimentation and with the Declaration of Helsinki of 1975, as revised in 2013. All participants were informed of the study's objectives and the anonymity of their responses before providing electronic informed consent.

## Supporting information

Supporting Information S1

## Data Availability

The data analysed during the current study are available from the corresponding author (via email) upon reasonable request.
