# Supplementary material for: Phase‐specific networks of job stress, anxiety and insomnia during acute crisis and chronic strain: A citywide study of public health nurses in Shanghai
Source: Gen Psychiatr. 2026 Sep 29;39(5):e70064. doi: 10.1002/gps3.70064 (PMC13620847; doi:10.1002/gps3.70064)
Supplement: Supplementary file 1 [file GPS3-39-e70064-s001.docx]

**Supplementary material for**

**Phase-specific networks of job stress, anxiety and insomnia during acute crisis and chronic strain: a citywide study of public health nurses in Shanghai**

**Supplementary tables**

**Table S1.** Scales and items used for the assessment of psychopathology symptoms.

**Table S2.** Psychological outcomes during the acute crisis and chronic strain phases.

**Table S3.** Regularised partial correlation (edge weight) matrices for the estimated psychometric network.

**Table S4.** Expected influence and bridge expected influence of network nodes during the acute crisis and chronic strain phases.

**Table S5.** Subgroup global network properties and Network Comparison Test results.

**Supplementary figures**

**Figure S1.** Estimation of node strength difference by bootstrapped difference test during different stages of the public health emergency.

**Figure S2.** Heatmap visualisations of the regularised partial correlation matrices across the acute crisis and chronic strain phases.

**Figure S3.** Edge weight differences during the acute crisis and chronic strain phases.

**Figure S4.** Bootstrapped confidence intervals of edge weights during the acute crisis phase.

**Figure S5.** Bootstrapped confidence intervals of edge weights during the chronic strain phase.

**Figure S6.** Directed acyclic graph depicting job stress, anxiety and insomnia among public health nurses across different phases of the public health emergency.

**Figure S7.** Psychometric networks by age subgroup across the acute crisis and chronic strain phases.

**Figure S8.** Psychometric networks by daily working hours during the acute crisis phase.

**Table S1.** Scales and items used for the assessment of psychopathology symptoms.

|  | **Symptoms** | | **Items** |
| --- | --- | --- | --- |
| **GAD-7** | Nervousness | | 1. Feeling nervous, anxious or on edge |
|  | Uncontrollable worry | | 2. Not being able to stop or control worrying |
|  | Excessive worry | | 3. Worrying too much about different things |
|  | Trouble relaxing | | 4. Trouble relaxing |
|  | Restlessness | | 5. Being so restless that it is hard to sit still |
|  | Irritability | | 6. Becoming easily annoyed or irritable |
|  | Feeling afraid | | 7. Feeling afraid as if something awful might happen |
| **JSQ-13** | Workload | Work demands efficiency | 1. Does your job often require you to work very quickly and efficiently? |
|  |  | Work demands effort | 2. Does your job often require you to work very hard? |
|  |  | Time pressure | 3. Do you often have to complete tasks within a very short period? |
|  |  | Handling important tasks | 4. Do you often take on very important tasks? |
|  |  | Increased workload | 5. Do you often feel that your workload has significantly increased? |
|  | Role conflict | Conflicting demands from peers | 6. Are you often asked by colleagues of a similar rank to perform conflicting tasks? |
|  |  | Conflicting responsibilities | 7. Are you often asked by others to perform tasks that conflict with your primary job responsibilities? |
|  | Role ambiguity | Unclear responsibilities | 8. Do you often feel uncertain about your job responsibilities? |
|  |  | Anticipating expectations | 9. Can you often anticipate others' expectations and demands regarding your work? |
|  |  | Clear performance standards | 10. Are your performance standards often clearly defined? |
|  | Skill utilisation | Application of academic knowledge | 11. Does your job often allow you to apply the knowledge and skills you learned in school or training? |
|  |  | Opportunities to excel | 12. Do you often get work opportunities that allow you to do things you are very good at? |
|  |  | Application of previous experience | 13. Do you often get to use the skills you have acquired from past experience and training? |
| **Insomnia** | Difficulty initiating sleep | | 1. I have difficulty falling asleep at night. |
|  | Difficulty maintaining sleep | | 2. I wake up during the night after having fallen asleep. |
|  | Early-morning awakening | | 3. I wake up early in the morning and have difficulty going back to sleep. |

Notes: GAD-7, 7-item Generalized Anxiety Disorder; JSQ-13, 13-item Job Stress Questionnaire.

#### **Table S2.** Psychological outcomes during the acute crisis and chronic strain phases.

|  | **Acute crisis phase (n = 1565)** | **Chronic strain phase (n = 1718)** | ***t* value** | ***df*** | ***P* value** |
| --- | --- | --- | --- | --- | --- |
| **GAD-7 Scale, Mean (SD)** |  |  |  |  |  |
| Nervousness | 0.93 (0.78) | 0.72 (0.71) | 7.96 | 3161.9 | < 0.001 |
| Uncontrollable worry | 0.82 (0.82) | 0.63 (0.72) | 7.15 | 3138.8 | < 0.001 |
| Excessive worry | 0.93 (0.79) | 0.73 (0.73) | 7.59 | 3181.1 | < 0.001 |
| Trouble relaxing | 0.90 (0.81) | 0.67 (0.73) | 8.57 | 3152.0 | < 0.001 |
| Restlessness | 0.69 (0.79) | 0.53 (0.70) | 6.07 | 3145.7 | < 0.001 |
| Irritability | 0.89 (0.79) | 0.68 (0.72) | 7.93 | 3166.9 | < 0.001 |
| Feeling afraid | 0.70 (0.80) | 0.51 (0.71) | 7.20 | 3143.9 | < 0.001 |
| Total score of GAD-7 | 5.87 (5.15) | 4.47 (4.61) | 8.15 | 3150.7 | < 0.001 |
| **JSQ-13 Scale, Mean (SD)** |  |  |  |  |  |
| Work demands efficiency | 4.15 (1.45) | 3.76 (1.43) | 7.66 | 3243.9 | < 0.001 |
| Work demands effort | 3.93 (1.48) | 3.40 (1.42) | 10.47 | 3226.8 | < 0.001 |
| Time pressure | 3.97 (1.47) | 3.58 (1.43) | 7.60 | 3233.6 | < 0.001 |
| Handling important tasks | 3.71 (1.44) | 3.36 (1.35) | 7.21 | 3202.5 | < 0.001 |
| Increased workload | 3.92 (1.51) | 3.36 (1.48) | 10.67 | 3240.5 | < 0.001 |
| Conflicting demands from peers | 2.88 (1.65) | 2.69 (1.56) | 3.42 | 3212.5 | 0.001 |
| Conflicting responsibilities | 2.88 (1.68) | 2.66 (1.58) | 3.88 | 3204.0 | < 0.001 |
| Unclear responsibilities | 2.18 (1.78) | 2.14 (1.69) | 0.77 | 3213.4 | 0.442 |
| Anticipating expectations | 3.43 (1.38) | 3.25 (1.35) | 3.78 | 3240.2 | < 0.001 |
| Clear performance standards | 3.44 (1.40) | 3.32 (1.31) | 2.50 | 3204.2 | 0.013 |
| Application of academic knowledge | 3.60 (1.31) | 3.46 (1.31) | 3.07 | 3250.4 | 0.002 |
| Opportunities to excel | 3.42 (1.29) | 3.35 (1.27) | 1.47 | 3239.9 | 0.142 |
| Application of previous experience | 3.62 (1.22) | 3.52 (1.25) | 2.49 | 3263.9 | 0.013 |
| Total score of JSQ-13 | 45.13 (13.34) | 41.84 (13.12) | 7.11 | 3241.8 | < 0.001 |
| **Insomnia, Mean (SD)** |  |  |  |  |  |
| Difficulty initiating sleep | 1.80 (0.63) | 1.68 (0.61) | 5.55 | 3230.0 | < 0.001 |
| Difficulty maintaining sleep | 1.84 (0.63) | 1.69 (0.62) | 6.69 | 3240.9 | < 0.001 |
| Early-morning awakening | 1.82 (0.65) | 1.68 (0.63) | 6.06 | 3232.5 | < 0.001 |

**Notes:** GAD-7, 7-item Generalized Anxiety Disorder; JSQ-13, 13-item Job Stress Questionnaire; SD, standard deviation.

**Table S3.** Regularised partial correlation (edge weight) matrices for the estimated psychometric network.

|  | **GAD1** | **GAD2** | **GAD3** | **GAD4** | **GAD5** | **GAD6** | **GAD7** | **JSQ1** | **JSQ2** | **JSQ3** | **JSQ4** | **JSQ5** | **JSQ6** | **JSQ7** | **JSQ8** | **JSQ9** | **JSQ10** | **JSQ11** | **JSQ12** | **JSQ13** | **DIS** | **DMS** |
| --- | --- | --- | --- | --- | --- | --- | --- | --- | --- | --- | --- | --- | --- | --- | --- | --- | --- | --- | --- | --- | --- | --- |
| **Acute crisis phase** | | | | | | | | | | | | | | | | | | | | | | |
| GAD1 |  |  |  |  |  |  |  |  |  |  |  |  |  |  |  |  |  |  |  |  |  |  |
| GAD2 | 0.309 |  |  |  |  |  |  |  |  |  |  |  |  |  |  |  |  |  |  |  |  |  |
| GAD3 | 0.233 | 0.226 |  |  |  |  |  |  |  |  |  |  |  |  |  |  |  |  |  |  |  |  |
| GAD4 | 0.169 | 0.179 | 0.273 |  |  |  |  |  |  |  |  |  |  |  |  |  |  |  |  |  |  |  |
| GAD5 | 0 | 0.216 | 0 | 0.110 |  |  |  |  |  |  |  |  |  |  |  |  |  |  |  |  |  |  |
| GAD6 | 0.169 | 0.049 | 0.183 | 0.125 | 0.184 |  |  |  |  |  |  |  |  |  |  |  |  |  |  |  |  |  |
| GAD7 | 0.047 | 0.060 | 0.043 | 0.074 | 0.413 | 0.160 |  |  |  |  |  |  |  |  |  |  |  |  |  |  |  |  |
| JSQ1 | 0 | 0 | 0 | 0 | -0.032 | 0 | -0.017 |  |  |  |  |  |  |  |  |  |  |  |  |  |  |  |
| JSQ2 | 0 | 0 | 0.006 | 0.007 | 0 | 0.011 | 0 | 0.378 |  |  |  |  |  |  |  |  |  |  |  |  |  |  |
| JSQ3 | 0 | 0 | 0 | 0 | -0.004 | 0 | 0 | 0.242 | 0.285 |  |  |  |  |  |  |  |  |  |  |  |  |  |
| JSQ4 | 0 | 0 | 0 | 0 | 0 | 0 | 0 | 0.159 | 0.130 | 0.303 |  |  |  |  |  |  |  |  |  |  |  |  |
| JSQ5 | 0.045 | 0 | 0 | 0.029 | -0.019 | 0 | 0 | 0.029 | 0.281 | 0.247 | 0.067 |  |  |  |  |  |  |  |  |  |  |  |
| JSQ6 | 0 | 0.016 | 0 | 0 | 0 | 0 | 0.003 | 0 | 0.016 | 0 | 0.081 | 0.023 |  |  |  |  |  |  |  |  |  |  |
| JSQ7 | 0 | 0 | 0 | 0 | 0.029 | 0 | 0 | 0 | 0 | 0 | 0.019 | 0.147 | 0.642 |  |  |  |  |  |  |  |  |  |
| JSQ8 | 0 | 0 | 0 | 0 | 0.080 | -0.003 | 0.071 | -0.089 | 0 | 0 | 0 | -0.050 | 0.186 | 0.290 |  |  |  |  |  |  |  |  |
| JSQ9 | 0 | 0 | 0 | 0 | 0 | 0 | 0 | 0.088 | 0.001 | 0 | 0.091 | 0.012 | 0.049 | 0.025 | 0.087 |  |  |  |  |  |  |  |
| JSQ10 | 0 | 0 | 0 | 0 | 0 | 0 | 0 | 0 | 0 | 0.014 | 0.032 | 0.039 | 0.005 | 0.013 | 0 | 0.357 |  |  |  |  |  |  |
| JSQ11 | 0 | 0 | 0 | 0 | 0 | 0 | 0 | 0.035 | 0.009 | 0 | 0 | 0 | -0.013 | 0 | 0 | 0.053 | 0.144 |  |  |  |  |  |
| JSQ12 | 0 | -0.002 | -0.009 | -0.015 | 0 | 0 | 0.007 | -0.013 | 0 | 0 | 0 | -0.006 | 0.002 | 0 | 0.038 | 0.089 | 0.121 | 0.223 |  |  |  |  |
| JSQ13 | -0.015 | -0.005 | 0 | 0 | 0 | -0.007 | 0 | 0.030 | 0 | 0 | 0.055 | 0 | 0 | 0 | -0.080 | 0.087 | 0.080 | 0.355 | 0.523 |  |  |  |
| DIS | 0.011 | 0 | 0.066 | 0.058 | 0.013 | 0 | 0 | 0 | 0 | 0 | 0.009 | 0 | 0 | 0 | 0 | 0 | 0 | 0 | 0 | 0 |  |  |
| DMS | 0 | 0.014 | 0.011 | 0 | 0 | 0.051 | 0.004 | 0 | 0 | 0 | 0 | 0 | 0.019 | 0 | 0 | -0.025 | 0 | 0 | 0 | 0 | 0.384 |  |
| EMA | 0.020 | 0.005 | 0.001 | 0 | 0.020 | 0.005 | 0.066 | 0 | 0 | 0 | 0 | 0.031 | 0 | 0 | 0 | 0 | 0 | 0 | 0 | -0.011 | 0.322 | 0.363 |
| **Chronic strain phase** | | | | | | | | | | | | | | | | | | | | | | |
| GAD1 |  |  |  |  |  |  |  |  |  |  |  |  |  |  |  |  |  |  |  |  |  |  |
| GAD2 | 0.202 |  |  |  |  |  |  |  |  |  |  |  |  |  |  |  |  |  |  |  |  |  |
| GAD3 | 0.284 | 0.214 |  |  |  |  |  |  |  |  |  |  |  |  |  |  |  |  |  |  |  |  |
| GAD4 | 0.194 | 0.232 | 0.245 |  |  |  |  |  |  |  |  |  |  |  |  |  |  |  |  |  |  |  |
| GAD5 | 0.019 | 0.148 | 0 | 0.156 |  |  |  |  |  |  |  |  |  |  |  |  |  |  |  |  |  |  |
| GAD6 | 0.123 | 0.023 | 0.144 | 0.198 | 0.172 |  |  |  |  |  |  |  |  |  |  |  |  |  |  |  |  |  |
| GAD7 | 0.029 | 0.155 | 0.058 | 0.027 | 0.459 | 0.156 |  |  |  |  |  |  |  |  |  |  |  |  |  |  |  |  |
| JSQ1 | 0 | 0 | 0 | 0 | -0.018 | 0 | -0.017 |  |  |  |  |  |  |  |  |  |  |  |  |  |  |  |
| JSQ2 | 0 | 0 | 0 | 0 | 0 | 0 | 0 | 0.341 |  |  |  |  |  |  |  |  |  |  |  |  |  |  |
| JSQ3 | 0 | 0 | 0 | 0.004 | 0 | 0 | 0 | 0.239 | 0.295 |  |  |  |  |  |  |  |  |  |  |  |  |  |
| JSQ4 | 0 | 0 | 0 | 0 | 0 | 0 | 0 | 0.121 | 0.173 | 0.292 |  |  |  |  |  |  |  |  |  |  |  |  |
| JSQ5 | 0 | 0 | 0.013 | 0 | 0 | 0.036 | 0 | 0.058 | 0.214 | 0.168 | 0.122 |  |  |  |  |  |  |  |  |  |  |  |
| JSQ6 | 0 | 0 | 0 | 0 | 0 | 0 | 0.010 | 0 | 0.024 | 0 | 0.049 | 0.123 |  |  |  |  |  |  |  |  |  |  |
| JSQ7 | 0 | 0.001 | 0 | 0 | 0 | 0.010 | 0.017 | 0 | 0.035 | 0 | 0.014 | 0.133 | 0.680 |  |  |  |  |  |  |  |  |  |
| JSQ8 | 0 | 0.004 | 0 | 0 | 0.045 | 0 | 0.043 | -0.102 | 0 | -0.001 | 0 | -0.048 | 0.205 | 0.262 |  |  |  |  |  |  |  |  |
| JSQ9 | 0 | 0 | 0 | 0 | 0 | 0 | 0 | 0.035 | 0 | 0 | 0.139 | 0.076 | 0 | 0.031 | 0.047 |  |  |  |  |  |  |  |
| JSQ10 | 0 | 0 | 0 | 0 | 0 | 0 | -0.005 | 0.019 | 0.007 | 0.019 | 0.017 | 0.044 | 0 | 0 | 0.018 | 0.365 |  |  |  |  |  |  |
| JSQ11 | 0 | -0.005 | 0 | 0 | 0 | 0 | 0 | 0.013 | 0 | 0 | 0 | 0 | 0 | -0.010 | 0 | 0.086 | 0.139 |  |  |  |  |  |
| JSQ12 | 0 | 0 | -0.003 | 0 | 0 | 0 | 0 | 0 | -0.004 | 0 | 0.030 | -0.025 | 0 | 0 | 0.050 | 0.066 | 0.079 | 0.230 |  |  |  |  |
| JSQ13 | 0 | 0 | 0 | -0.007 | -0.019 | 0 | -0.004 | 0.060 | 0 | 0 | 0.011 | 0 | 0 | 0 | -0.070 | 0.064 | 0.087 | 0.397 | 0.527 |  |  |  |
| DIS | 0.018 | 0.023 | 0.017 | 0.026 | 0 | 0.045 | 0 | 0 | 0.007 | 0.001 | 0 | 0.005 | 0 | 0 | 0 | 0 | 0 | 0 | 0 | 0 |  |  |
| DMS | 0 | 0.007 | 0.003 | 0.039 | 0.017 | 0 | 0 | 0 | 0 | 0 | 0 | 0 | 0 | 0.020 | 0 | 0 | 0 | 0 | 0 | -0.012 | 0.345 |  |
| EMA | 0 | 0.013 | 0.055 | 0 | 0.013 | 0.011 | 0 | 0 | 0 | 0 | 0 | 0.040 | 0.015 | 0.001 | 0.015 | 0 | 0 | 0 | 0 | -0.009 | 0.305 | 0.406 |

**Notes:** Values represent regularised partial correlation coefficients estimated via the Extended Bayesian Information Criterion Graphical Least Absolute Shrinkage and Selection Operator (EBICglasso). To improve readability while preserving full transparency, only the lower triangle of each symmetric matrix is presented; diagonal elements are zero by definition and upper-triangular elements are redundant. Following the regularisation procedure inherent to EBICglasso, negligible partial correlations are shrunk exactly to zero to reduce spurious associations and enhance interpretability.^1–3^ Consequently, the matrices are expectedly sparse, with non-zero edges predominantly concentrated within symptom domains.

**Abbreviations:** DIS, difficulty initiating sleep (insomnia); DMS, difficulty maintaining sleep (insomnia); EMA, early-morning awakening (insomnia); GAD1, nervousness (anxiety); GAD2, uncontrollable worry (anxiety); GAD3, excessive worry (anxiety); GAD4, trouble relaxing (anxiety); GAD5, restlessness (anxiety); GAD6, irritability (anxiety); GAD7, feeling afraid (anxiety); JSQ1, work demands efficiency (job stress); JSQ2, work demands effort (job stress); JSQ3, time pressure (job stress); JSQ4, handling important tasks (job stress); JSQ5, increased workload (job stress); JSQ6, conflicting demands from peers (job stress); JSQ7, conflicting responsibilities (job stress); JSQ8, unclear responsibilities (job stress); JSQ9, anticipating expectations (job stress); JSQ10, clear performance standards (job stress); JSQ11, application of academic knowledge (job stress); JSQ12, opportunities to excel (job stress); JSQ13, application of previous experience (job stress).

**Table S4.** Expected influence and bridge expected influence of network nodes during the acute crisis and chronic strain phases.

| **Node** | **Item name** | **Acute crisis phase** | | **Chronic strain phase** | |
| --- | --- | --- | --- | --- | --- |
|  |  | **EI** | **BEI** | **EI** | **BEI** |
| GAD-7 Scale | | | | | |
| GAD1 | Nervousness | 0.988 | 0.061 | 0.869 | 0.018 |
| GAD2 | Uncontrollable worry | 1.066 | 0.027 | 1.016 | 0.043 |
| GAD3 | Excessive worry | 1.033 | 0.075 | 1.030 | 0.085 |
| GAD4 | Trouble relaxing | 1.010 | 0.079 | 1.113 | 0.061 |
| GAD5 | Restlessness | 1.010 | 0.087 | 0.991 | 0.038 |
| GAD6 | Irritability | 0.927 | 0.058 | 0.918 | 0.102 |
| GAD7 | Feeling afraid | 0.930 | 0.133 | 0.929 | 0.045 |
| JSQ-13 Scale | | | | | |
| JSQ1 | Work demands efficiency | 0.809 | -0.049 | 0.749 | -0.035 |
| JSQ2 | Work demands effort | 1.123 | 0.024 | 1.092 | 0.007 |
| JSQ3 | Time pressure | 1.087 | -0.004 | 1.017 | 0.005 |
| JSQ4 | Handling important tasks | 0.945 | 0.009 | 0.968 | 0 |
| JSQ5 | Increased workload | 0.876 | 0.087 | 0.961 | 0.095 |
| JSQ6 | Conflicting demands from peers | 1.030 | 0.038 | 1.105 | 0.024 |
| JSQ7 | Conflicting responsibilities | 1.164 | 0.029 | 1.194 | 0.049 |
| JSQ8 | Unclear responsibilities | 0.529 | 0.148 | 0.468 | 0.107 |
| JSQ9 | Anticipating expectations | 0.914 | -0.025 | 0.910 | 0 |
| JSQ10 | Clear performance standards | 0.804 | 0 | 0.790 | -0.005 |
| JSQ11 | Application of academic knowledge | 0.806 | 0 | 0.850 | -0.005 |
| JSQ12 | Opportunities to excel | 0.957 | -0.020 | 0.951 | -0.003 |
| JSQ13 | Application of previous experience | 1.013 | -0.037 | 1.026 | -0.051 |
| Insomnia | | | | | |
| DIS | Difficulty initiating sleep | 0.863 | 0.157 | 0.791 | 0.141 |
| DMS | Difficulty maintaining sleep | 0.820 | 0.073 | 0.825 | 0.075 |
| EMA | Early-morning awakening | 0.821 | 0.136 | 0.864 | 0.153 |

**Notes:** BEI, bridge expected influence; DIS, difficulty initiating sleep; DMS, difficulty maintaining sleep; EI, expected influence; EMA, early-morning awakening; GAD-7, 7-item Generalized Anxiety Disorder; JSQ-13, 13-item Job Stress Questionnaire.

**Table S5.** Subgroup global network properties and Network Comparison Test results.

| Subgroup | N | Nodes | Non-zero edges | Density | Clustering coefficient | Average path length | NCT structure (M, P) | NCT strength  (S, P) |
| --- | --- | --- | --- | --- | --- | --- | --- | --- |
| Acute crisis phase |  |  |  |  |  |  |  |  |
| Age < 36 years | 733 | 23 | 112 | 0.443 | 0.556 | 20.790 | 0.160, 0.502 | 0.692, 0.021 |
| Age ≥ 36 years | 832 | 23 | 103 | 0.407 | 0.585 | 24.960 | — | — |
| Work hours ≤ 8 h/day | 1099 | 23 | 114 | 0.451 | 0.595 | 22.975 | 0.182, 0.374 | 0.359, 0.261 |
| Work hours > 8 h/day | 466 | 23 | 89 | 0.352 | 0.566 | 22.042 | — | — |
| Chronic strain phase | | | | | | | | |
| Age < 36 years | 732 | 23 | 113 | 0.447 | 0.623 | 25.212 | 0.182, 0.369 | 0.116, 0.686 |
| Age ≥ 36 years | 986 | 23 | 131 | 0.518 | 0.616 | 25.756 | — | — |

**Notes:** M, maximum edge-weight difference statistic used in the network structure invariance test; NCT, Network Comparison Test (1000 permutations); S, global strength difference statistic used in the global strength invariance test. NCT statistics are reported for the pairwise comparison within each subgroup category (age or work hours) and phase; "—" indicates the reference group in the pairwise comparison.

**Figure S1.** Estimation of node strength difference by bootstrapped difference test during different stages of the public health emergency.

**Notes:** DIS, difficulty initiating sleep; DMS, difficulty maintaining sleep; EMA, early-morning awakening; GAD-7, 7-item Generalized Anxiety Disorder; JSQ-13, 13-item Job Stress Questionnaire. Bootstrapped difference tests between node strength of the 23 nodes. Grey boxes represent nodes that do not significantly differ from one another. Black boxes represent significant differences. White boxes represent the values of node strength.

**Figure S2.** Heatmap visualisations of the regularised partial correlation matrices across the acute crisis and chronic strain phases.

**Notes:** Colour intensity and hue indicate the magnitude and direction of regularised partial correlations (red = positive, blue = negative, white = zero midpoint). Values shrunk to exactly zero by the EBICglasso regularisation procedure are displayed in light grey to distinguish absent edges from the background. Nodes are ordered by symptom domain: anxiety (GAD1–GAD7), job stress (JSQ1–JSQ13) and insomnia (DIS–EMA). Abbreviations: DIS, difficulty initiating sleep; EBICglasso, Extended Bayesian Information Criterion Graphical Least Absolute Shrinkage and Selection Operator; EMA, early-morning awakening; GAD1–GAD7, individual items of the 7-item Generalized Anxiety Disorder scale; JSQ1–JSQ13, individual items of the 13-item Job Stress Questionnaire.

**Figure S3.** Edge weight differences during the acute crisis and chronic strain phases.

**Notes:** DIS, difficulty initiating sleep; DMS, difficulty maintaining sleep; EMA, early-morning awakening; GAD-7, 7-item Generalized Anxiety Disorder; JSQ-13, 13-item Job Stress Questionnaire. Edges that differ significantly from each other are depicted by black boxes.

**Figure S4.** Bootstrapped confidence intervals of edge weights during the acute crisis phase.

**Notes:** DIS, difficulty initiating sleep; DMS, difficulty maintaining sleep; EMA, early-morning awakening; GAD-7, 7-item Generalized Anxiety Disorder; JSQ-13, 13-item Job Stress Questionnaire. Red dots indicate the estimated values of each edge weight, ordered from highest to lowest. Grey bands represent the 95% confidence intervals of edge weights estimated using a non-parametric bootstrap procedure. Wider intervals indicate lower estimation stability, whereas narrower intervals indicate higher stability.

**Figure S5.** Bootstrapped confidence intervals of edge weights during the chronic strain phase.

**Notes:** DIS, difficulty initiating sleep; DMS, difficulty maintaining sleep; EMA, early-morning awakening; GAD-7, 7-item Generalized Anxiety Disorder; JSQ-13, 13-item Job Stress Questionnaire. Red dots indicate the estimated values of each edge weight, ordered from highest to lowest. Grey bands represent the 95% confidence intervals of edge weights estimated using a non-parametric bootstrap procedure. Wider intervals indicate lower estimation stability, whereas narrower intervals indicate higher stability.

**Figure S6.** Directed acyclic graph depicting job stress, anxiety and insomnia among public health nurses across different phases of the public health emergency.

**Notes:** DIS, difficulty initiating sleep (insomnia); DMS, difficulty maintaining sleep (insomnia); EMA, early-morning awakening (insomnia); GAD1, nervousness (anxiety); GAD2, uncontrollable worry (anxiety); GAD3, excessive worry (anxiety); GAD4, trouble relaxing (anxiety); GAD5, restlessness (anxiety); GAD6, irritability (anxiety); GAD7, feeling afraid (anxiety); JSQ1, work demands efficiency (job stress); JSQ2, work demands effort (job stress); JSQ3, time pressure (job stress); JSQ4, handling important tasks (job stress); JSQ5, increased workload (job stress); JSQ6, conflicting demands from peers (job stress); JSQ7, conflicting responsibilities (job stress); JSQ8, unclear responsibilities (job stress); JSQ9, anticipating expectations (job stress); JSQ10, clear performance standards (job stress); JSQ11, application of academic knowledge (job stress); JSQ12, opportunities to excel (job stress); JSQ13, application of previous experience (job stress).

**Figure S7.** Psychometric networks by age subgroup across the acute crisis and chronic strain phases.

**Notes:** Panels A and B display the estimated EBICglasso networks for younger (< 36 years) and older (≥ 36 years) nurses during the acute crisis phase (n = 733 and 832, respectively). Panels C and D display the corresponding networks during the chronic strain phase (n = 732 and 986, respectively). Nodes represent individual questionnaire items: orange = anxiety (GAD-7), green = job stress (JSQ-13), blue = insomnia (DIS, DMS, EMA). Edge thickness and colour saturation indicate the absolute magnitude of regularised partial correlations; blue edges denote positive associations and red dashed edges denote negative associations. Node size reflects strength centrality.

**Abbreviations:** DIS, difficulty initiating sleep (insomnia); DMS, difficulty maintaining sleep (insomnia); EBICglasso, Extended Bayesian Information Criterion Graphical Least Absolute Shrinkage and Selection Operator; EMA, early-morning awakening (insomnia); GAD-7, 7-item Generalized Anxiety Disorder; GAD1, nervousness (anxiety); GAD2, uncontrollable worry (anxiety); GAD3, excessive worry (anxiety); GAD4, trouble relaxing (anxiety); GAD5, restlessness (anxiety); GAD6, irritability (anxiety); GAD7, feeling afraid (anxiety); JSQ-13, 13-item Job Stress Questionnaire; JSQ1, work demands efficiency (job stress); JSQ2, work demands effort (job stress); JSQ3, time pressure (job stress); JSQ4, handling important tasks (job stress); JSQ5, increased workload (job stress); JSQ6, conflicting demands from peers (job stress); JSQ7, conflicting responsibilities (job stress); JSQ8, unclear responsibilities (job stress); JSQ9, anticipating expectations (job stress); JSQ10, clear performance standards (job stress); JSQ11, application of academic knowledge (job stress); JSQ12, opportunities to excel (job stress); JSQ13, application of previous experience (job stress).

**Figure S8.** Psychometric networks by daily working hours during the acute crisis phase.

**Notes:** Panel A displays the estimated EBICglasso network for nurses working ≤ 8 hours per day (n = 1099) and Panel B for those working > 8 hours per day (n = 466) during the acute crisis phase. Nodes represent individual questionnaire items: orange = anxiety (GAD-7), green = job stress (JSQ-13), blue = insomnia (DIS, DMS, EMA). Edge thickness and colour saturation indicate the absolute magnitude of regularised partial correlations; blue edges denote positive associations and red dashed edges denote negative associations. Node size reflects strength centrality. Owing to insufficient sample size in the > 8 hours group during the chronic strain phase (n = 106), which was below the commonly recommended minimum of five to ten observations per estimated node for exploratory EBICglasso network estimation, working-hours subgroup analysis was restricted to the acute phase. Abbreviations: DIS, difficulty initiating sleep; DMS, difficulty maintaining sleep; EBICglasso, Extended Bayesian Information Criterion Graphical Least Absolute Shrinkage and Selection Operator; EMA, early-morning awakening; GAD-7, 7-item Generalized Anxiety Disorder; JSQ-13, 13-item Job Stress Questionnaire.

**References**

1.	Epskamp S, Borsboom D, Fried EI. Estimating psychological networks and their accuracy: A tutorial paper. Behav Res Methods. 2018;50(1):195-212.

2.	Wang S, Hou W, Tao Y, Ma Z, Li K, Wang Y, et al. Mapping network connection among symptoms of anxiety, depression, and sleep disturbance in Chinese high school students. Front Public Health. 2022;10:1015166.

3.	Zhang Z, Chen H, Li X, Chen S, Wang Z, Yang J, et al. Differences in the depression and burnout networks between doctors and nurses: evidence from a network analysis. BMC Public Health. 2024;24(1):1668.
